# The Polish Version of the Avoidant/Restrictive Food Intake Disorder Questionnaire—Parents Report (ARFID-Q-PR) and the Nine Items Avoidant/Restrictive Food Intake Disorder Screen—Parents Report (NIAS-PR): Maternal Perspective

**DOI:** 10.3390/nu14153175

**Published:** 2022-08-02

**Authors:** Beata Ziółkowska, Jarosław Ocalewski, Hana Zickgraf, Anna Brytek-Matera

**Affiliations:** 1 Faculty of Psychology, Kazimierz Wielki University, 85-064 Bydgoszcz, Poland; beata.ziolkowska@ukw.edu.pl (B.Z.); jaroslaw.ocalewski@ukw.edu.pl (J.O.); 2 Department of Psychology, University of South Alabama, Mobile, AL 36688, USA; zickgraf@southalabama.edu; 3 Institute of Psychology, University of Wrocław, 50-527 Wrocław, Poland

**Keywords:** avoidant/restrictive food intake disorder, feeding disorders, children, developmental disorders, maternal perspective

## Abstract

The aim of the present study was to develop and validate the Avoidant/Restrictive Food Intake Disorder Questionnaire—Parents Report (ARFID-Q-PR), a new tool to diagnose ARFID, based on a report submitted by Polish mothers of children aged 2 to 10 years. In total, 167 mothers of boys and girls aged 2 to 10 participated in the study. We used the ARFID-Q-PR and the Nine Items Avoidant/Restrictive Food Intake Disorder Screen—Parents Report (NIAS-PR). In addition, all mothers were asked to provide information on age, sex, height and weight, chronic somatic diseases, neurodevelopmental and mental disorders as well as intellectual disability of their children. Results of the reliability analysis demonstrated that the ARFID-Q-PR had adequate internal consistency (Cronbach’s alpha of 0.84). The stability of the ARFID-Q-PR factorial structure was confirmed. It is composed of three subscales: (1) attitudes to food; (2) justification for restrictions; (3) somatic symptoms. Our findings demonstrated that the ARFID-Q-PR total score was positively associated with the NIAS-PR total score. In addition, children with developmental and mental disorders substantially demonstrated more ARFID symptoms than did the children in the general population. The Polish version ARFID-Q-PR can be used to recognize the ARFID symptoms in young children by the main feeder in the family—mother or father.

## 1. Introduction

In the Diagnostic and Statistical Manual of Mental Disorders, Fifth Edition (DSM-5) [1], a new disorder has been included—Avoidant/Restrictive Food Intake Disorder (ARFID). No large epidemiological studies have yet been performed that would allow for a reliable assessment of the frequency of ARFID occurrence. ARFID was added to the DSM-5 and the International Classification of Diseases, Tenth Edition (ICD-10) in response to evident clinical need: eating disorder specialists and other clinicians reported that patients were presenting for treatment of eating disorder or somatic symptoms with patterns of restrictive eating not consistent with the diagnoses of anorexia nervosa or bulimia nervosa or fully explained by organic illness or developmental disorders; many of these patients were younger than is typical for the onset of restrictive eating disorder [2,3]. The core diagnostic symptoms of ARFID, weight loss, nutritional deficiencies, dependence on supplements for adequate nutrition or calories, and/or psychosocial impairment, have heterogeneous causes [4], with diagnostic criteria excluding only eating restrictions caused by overvaluation of weight and shape, body dysmorphia, and fear of weight gain [1]. Three patterns of ARFID restrictive eating have consistently been described, in both community and clinical samples: selective/neophobic (i.e., “picky” eating), poor appetite or limited interest in food, and fear of aversive consequences from eating [5,6,7]. Although largescale population prevalence estimates are not available, ARFID has been shown to be common in both community and clinical pediatric samples [5,8,9]. The short history of research on ARFID provides important descriptive data, but it is still insufficient for understanding its specifics and planning effective forms of treatment to patients and their families. Currently, there are many tools in the world for assessing eating disorders. Below are some that have been specifically designed to evaluate ARFID as a conceptualization of the current DSM/ICD (Table 1).

Although the existence of distinct patterns of restrictive eating that drive ARFID symptoms is well-documented, there are currently no diagnostic specifiers for ARFID [1], potentially leading to heterogeneity in the descriptive psychopathology literature. While some researchers lean towards the concept of distinct manifestations of ARFID [11], others [4,12] have argued that combined presentations are the norm, although eating restrictions can occur independently, and one presentation (e.g., selective/neophobic eating, appetite/interest, fear) can usually be identified as the primary contributor to presenting symptoms. ARFID is therefore a heterogeneous disorder, as its clinical picture is quite varied in terms of the quality and intensity of symptoms in different patients and ranges from long-term radical food selection (especially the elimination of solid food) to refusing to eat due to a traumatic incident involving a specific food (e.g., choking on a chicken bone) or vomiting (e.g., in reaction to food poisoning) [4,13,14].

### 1.1. ARFID Incidence, Prevalence, and Diagnostic Considerations

Although ARFID can onset at any age or developmental stage, ARFID patients presenting for eating disorder-focused treatment tend to be younger than their counterparts with other eating disorders, despite often having a longer illness duration [3,15,16]. Some presentations of ARFID, particularly the selective/neophobic presentation, almost invariably begin early in development, becoming increasingly impairing when they persist beyond the normative stage of neophobia, which occurs at the age of 2 to 6 [10,17]. Indeed, one largescale epidemiological study from Canada found that older children with ARFID generally had more severe medical and psychological morbidity than younger children [8]. Common comorbidities of ARFID include neurodevelopmental disorders (e.g., autism), sensory integration disorders, alexithymia, intellectual disability, difficult temperament, high levels of externalization and internalization, psychomotor hyperactivity, anxiety disorders, obsessive-compulsive disorders, depressive disorders, suicide attempts and self-harm [13,15,18,19,20,21,22]. It is also not uncommon for ARFID to develop from childhood health problems that lead to gastrointestinal disturbances [9,23,24,25]. In such a case, the disorder results most often from the fear of negative somatic consequences of food intake (e.g., pain) and may be maintained by inadvertent negative reinforcement of disruptive mealtime behaviors by caregivers [25,26]. A restrictive diet, however, exacerbates problems related to gastric motility, and regular eating becomes an increasing challenge for the patient [4].

Retrospective chart reviews in pediatric medical settings, including eating disorder services, derived from patients seen before the introduction of the ARFID diagnosis in 2013, report a range of prevalence estimates from 1.5 to 32% of respondents so far [2,3,5,15,27,28,29,30,31]. This significant discrepancy in the results is primarily due to the fact that the research involved both groups of previously undiagnosed people and people with various diagnoses, including mental disorders and somatic diseases. Reviews of medical charts after 2013 have found that 8–22% of children and adolescents seen as inpatients and outpatients at pediatric hospitals are diagnosed with ARFID [32,33]. The limited data from community samples of children in Japan, France, and Switzerland suggests an ARFID prevalence between 1–3.5% [30,34]. Despite the demonstrated differences in estimates, it can be assumed that ARFID is a disorder observed both in clinical settings and in the general population [4,20].

The DSM-5TR classification adopted the following diagnostic criteria for ARFID (Box 1).

Box 1 **Diagnostic criteria for ARFID (307.59) according to the Diagnostic and Statistical Manual of Mental Disorders, Fifth Edition, Text Revision (DSM-5TR).**

(A) An eating or feeding disturbance (e.g., apparent lack of interest in eating or food; avoidance based on the sensory characteristics of food; concern about aversive consequences of eating) associated with one (or more) of the following:
  - Significant weight loss (or failure to achieve expected weight gain or faltering growth in children).
  - Significant nutritional deficiency.
  - Dependence on enteral feeding or oral nutritional supplements.
  - Marked interference with psychosocial functioning.
(B) The disturbance is not better explained by lack of available food or by an associated culturally sanctioned practice.
(C) The eating disturbance does not occur exclusively during the course of anorexia nervosa or bulimia nervosa, and there is no evidence of a disturbance in the way in which one’s body weight or shape is experienced.
(D) The eating disturbance is not attributable to a concurrent medical condition or not better explained by another mental disorder. When the eating disturbance occurs in the context of another condition or disorder, the severity of the eating disturbance exceeds that routinely associated with the condition or disorder and warrants additional clinical attention.

In the process of differential diagnosis, when verifying the hypothesis of ARFID diagnosis, in addition to other abnormal eating behaviors (e.g., anorexia nervosa, orthorexia, nutritional neophobia), one should also take into account somatic diseases (including disorders of the digestive tract, food allergies, hormonal disorders, diabetes, neoplastic diseases, etc.), as well as other mental disorders including mood, anxiety, somaticizing, trauma-related, obsessive-compulsive, and other eating disorders [2,3].

Although we do not yet have longitudinal studies in the field of ARFID, it seems that problems in the patient’s affective functioning may be, on the one hand, a risk factor and, on the other hand, a consequence of this disorder [20,21,22]. It has been noted that the symptoms of emotional disorders increase with the escalation of ARFID symptoms, but it has also been proven that compared to patients with anorexia and bulimia nervosa, people with ARFID present with comparable levels of anxiety disorder symptomatology but less depression [15]. It has also been shown [18] that patients with an acute onset of ARFID are more prone to suicidal thoughts and/or self-harm than their peers with chronic onset, although as the authors note, these differences may be partially accounted for by differing presentations having both different patterns of onset and comorbidity, e.g., [6].

### 1.2. Objective of the Study

The assessment of ARFID closely depends on the quality of the measurement methods. In Poland, there is no measure of diagnosis of ARFID symptoms, neither in the form of a self-report (for older children, adolescents and adults), nor in the form of a parental report. Younger children are unable to fill out the self-report of ARFID symptoms and parent-reported instruments are therefore needed [34].

The overall aim of the study was to increase the availability of validated ARFID symptom measures for children/in Polish. The first objective was to develop a new measure—the Avoidant/Restrictive Food Intake Disorder Questionnaire—Parents Report (ARFID-Q-PR) and to evaluate its measurement properties.

Regarding the cultural adaptation, validation, and development of the questionnaire(s) there is a recommendation for the use of the Consensus-based Standards for the selection of health Measurement Instruments (COSMIN) taxonomy of measurement properties of outcome measurement questionnaires [35]. Reliability, validity, and interpretability should be evaluated for the tool(s). We decided to compare the results of the ARFID-Q-PR with the results of the validated Nine Items Avoidant/Restrictive Food Intake Disorder Screen Parents Report (NIAS-PR) [36]. In addition, the second objective was to investigate the factorial structure of the Polish version of the NIAS-PR.

The NIAS and EDY-Q are methods used in the previous studies. In Poland, there is a lack of good psychometric instruments measuring ARFID. Therefore, there is a need to develop a Polish tool as well as translating the English-language tool. Further, the two instruments are designed to measure overlapping but different constructs. The NIAS was developed as a screening instrument that can identify the primary presentation(s) of restrictive eating causing ARFID symptoms [36,37]. The Polish tool assesses ARFID eating restrictions and the secondary somatic effects that may result from a poor diet.

## 2. Methods

### 2.1. Participants and Procedure

The research project was carried out at the turn of 2020 and 2021, partly online, due to the SARS-CoV-2 pandemic. The research was initially conducted in person, with recruitment in institutions such as nurseries, kindergartens, children’s clubs, and schools in Wielkopolska, Silesia, Lower Silesia, and Kujawsko-Pomorskie. Then—due to the epidemiological situation—an electronic version of the study was prepared using Google Forms, and a link to it was made available on online forums for parents from the same regions. In both recruitment procedures, caregivers were assured of the voluntary nature of participation and confidentiality of the research and provided informed consent.

The research project was approved by the Scientific Research Ethics Committee located at the Faculty of Psychology of the UKW in Bydgoszcz, Poland (consent no. 2/5.11.2019).

Answers were obtained from 167 mothers of children aged 2–10 years old, of which 18.56% (N = 31) had chronic illness and 81.44% (N = 136) were healthy. The share of girls and boys in the sample was similar and amounted to 49.10% (N = 82) and 50.90% (N = 85), respectively. The mean age of the examined children was 5.77 years (SD = 2.47), including boys 5.31 (SD = 2.33) and girls 6.25 (SD = 2.53).

### 2.2. Measures

#### 2.2.1. Demographic Schedule

The respondents were asked to answer questions concerning: the child’s date of birth, sex, height and weight, chronic somatic diseases, neurodevelopmental and mental disorders, and intellectual disability. BMI indices were calculated on the basis of data on the child’s height and weight, referring the result to age and gender, in accordance with the norms contained in percentile grids.

#### 2.2.2. Nine Items Avoidant/Restrictive Food Intake Disorder Screen—Parent Report (NIAS-PR)

Two tools were used in the present study. First—*Nine Items Avoidant/Restrictive Food Intake Disorder Screen—Parent Report* (NIAS-PR) developed in the United States [37.38]. The NIAS was originally developed in an adult sample, where the three-factor structure, high internal consistency, and excellent test-retest reliability were established [36]. The NIAS includes nine items rated on a 0–5 Likert scale from “Strongly disagree”, “disagree”, to “Strongly agree”. The NIAS has three factors, each with three items assessing the degree to which respondents endorse symptoms of selective-neophobic eating (items 1–3), poor appetite/limited interest in eating (items 4–6), and fear of aversive consequences (e.g., choking, vomiting, and pain from eating) (items 7–9). The NIAS has been formally validated in research and clinical settings [36].

The original version of the Nine Items Avoidant/Restrictive Food Intake Disorder Screen Parents Report has been translated into Polish for the current study (using a standard forward–backward translation procedure) (Table 2).

#### 2.2.3. ARFID Questionnaire—Parent Report (ARFID-Q-PR)

The second—Polish tool—proposed and developed by Ziółkowska, Ocalewski, and Brytek-Matera is called ARFID-Q-PR and was created in 2020. The construction of the ARFID-Q-PR consisted of four stages: test planning, test preparation, trial testing, and test evaluation [38]. In the first stage, the main construct and its symptoms were defined based on following the DSM-5 classification [1]. It was assumed that the ARFID-Q-PR aimed to provide information on the eating behavior of children from 2 to 10 years of age based on a report by their mothers. At the test preparation stage, 18 items were formulated regarding the key areas of the child’s functioning with regard to the definition of ARFID: 1. attitude to food—nutritional restrictions, 2. conditions for refusing to eat (sensory aversion, lack of interest in food, avoiding unpleasant consequences of eating), 3. somatic consequences of a poor diet. Mothers responded to each item on a five-point scale (“almost never”—”rarely”—”from time to time”—”often”—”almost always”). The list of items was then presented to three clinical psychologists who are specialized in development psychology and work with children. Their task was to indicate which group of given items belongs to the three domains of ARFID, and secondly, to rank them ranging from those that best described a given problem to those that characterized the specific area of the child’s functioning the least. After averaging the opinions of the competent judges, 14 items were selected for the final version of the tool (see Table 3). Then, 10 mothers of children aged 2 to 10 filled out the AFRID-Q-PR (the trial testing stage), and we collected comments on the comprehensibility of the test items. The evaluation stage was performed by examining 167 mothers of children with ARFID. The following measurement properties were defined: reliability (internal consistency), validity (criterion validity—concurrent validity), and interpretability [35]. To determine the concurrent validity, the results were compared with the validated Nine Items Avoidant/Restrictive Food Intake Disorder Screen Parents Report (NIAS-PR).

In the present study, the sample comprised only mothers, but it is intended for use by fathers or other caregivers responsible for preparing meals and feeding the children in the family. The authors also prepared a self-report version of the tool (ARFID-Q) for people from 11 years of age (research is currently underway).

Theoretically, three factors were distinguished: “attitude to food”, “justification of the restriction”, and “somatic condition”. The first one includes a group of statements describing the child’s behavior towards food; the second relates to the motives by which the child rejects the food or the conditions for which it takes it; the third, in turn, to the somatic effects of food restrictions, which may be the result of food restrictions and malnutrition.

## 3. Results

Statistical analyses were carried out using the STATICTICA 13, IBM SPSS 25, and IBM SPSS Amos programs. The significance level *p* was set at 0.05. Cronbach’s alpha was used to assess the internal consistency of the ARFID-Q-PR.

### 3.1. Factor Structure of Research Tools

To detect the ARFID-Q-PR factor structure, exploratory factor analysis (EFA) was first used, and the maximum likelihood method was adopted (the number of factors was based on the Kaiser criterion) [39]. Next, the authors used the standardized Varimax rotation method. To confirm the structure of the ARFID-Q-PR, confirmatory factor analysis (CFA) was performed. To evaluate the fit of the model, the following fit ratios were used with the respective cut-off values [40]. Root mean square error of approximation (RMSEA; less than 0.05 is a good fit) with 90% confidence interval, Confirmatory Fit Index (CFI; greater than 0.90—correct fit), Tucker–Lewis Index (TLI; greater than 0.95 indicates a good fit), the adjusted goodness of fit index AGFI, and the overall Chi square model (χ2). The analysis of the results began with checking the distributions of the variable “Eating avoidance/restriction disorder” as measured by the ARFID-Q- PR and the NIAS-PR tools. The obtained results did not confirm a normal distribution, but the values of skewness and kurtosis are in the range from −1 to 1, which allowed for the use of parametric tests [41]—Pearson’s r correlation (NIAS-PR: skewness = 0.79, kurtosis = 0.28; ARFID: skewness = 0.48, kurtosis = −0.30). Participants with missing data on the ARFID-Q-PR or validity measures were excluded (N = 9). Table 4 presents the results of the *r*-Pearson correlation between the ARFID-Q-PR and NIAS-PR subscales and metric variables.

The average score of the ARFID-Q-PR was 23.83, the lowest—13—and the highest—41 points. In the case of the NIAS-PR, these values were 20.4, 9, and 48, respectively. There were statistically significant correlations between the ARFID-Q-PR “attitude to food” and “justification for restriction” and sum of ARFID-Q-PR scores. There was no statistically significant correlation between the “somatic condition” subscale and the sum of ARFID-Q-PR (*r* = 0.14). It was proved that the sum of ARFID-Q-PR is statistically significantly correlated with the results of the NIAS-PR test (*r* = 0.62). Based on the results database, a factor analysis (EFA) was performed for ARFID-Q-PR (Table 5).

The analysis of the collected material allows—in line with the assumptions—to select three groups of factors/subscales (Table 6). Finally, five items were included in the “attitude to food” subscale (items: 1, 4, 10, 12, 13), four in the “justification for restriction” subscale (items: 2, 5, 8, 7), and five in the “health condition” subscale somatic” (items 3, 6, 9, 11, 14).

Then, the reliability of the entire ARFID-Q-PR test and its individual subscales was analyzed. The value of the Cronbach‘s Alpha index for the entire tool, based on a study of 167 mothers of children from 2 to 10 years old without a diagnosis of disorders, was satisfactory and amounted to 0.84. The reliability for individual subscales is presented in the table below (Table 7).

The reliability of the entire NIAS-PR test and its individual subscales was analyzed (Table 8).

### 3.2. Structural Modeling for Research Tools

**Figure 1:**
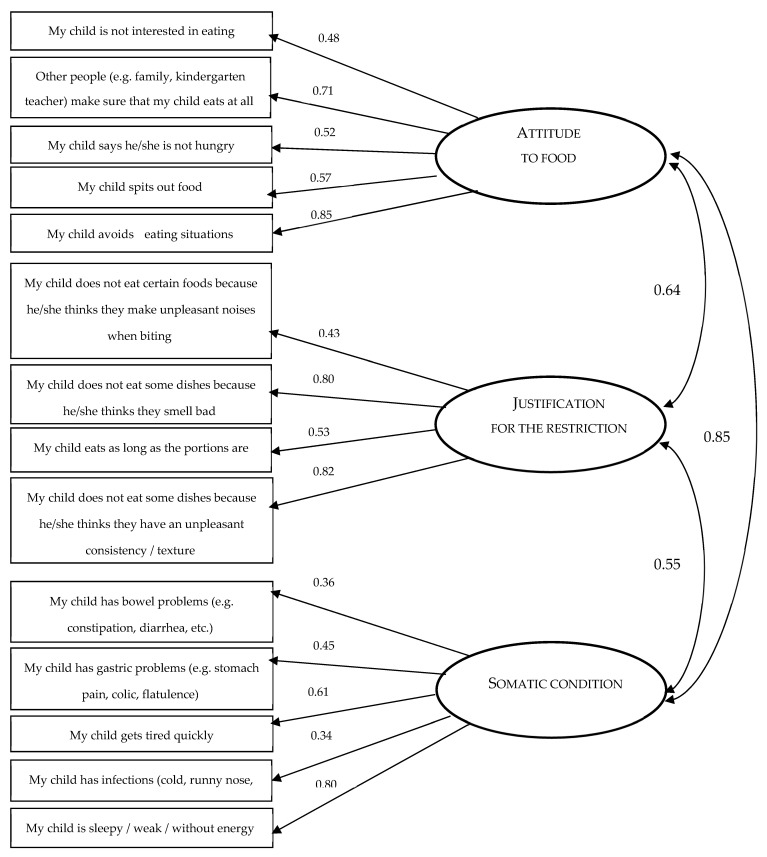
Confirmatory analysis showing the three factors of ARFID-Q-PR.

Structural modeling was then performed for a three-factor model. The confirmatory CFA analysis for ARFID-Q-PR confirmed that the theoretical model fits well with the variance–covariance matrix from the sample (Figure 1 and Table 9). The values of the CMIN, CMIN/df RMSEA, AGFI, TLI, CFI statistics indicate that the three-factor model is acceptable.

**Figure 2:**
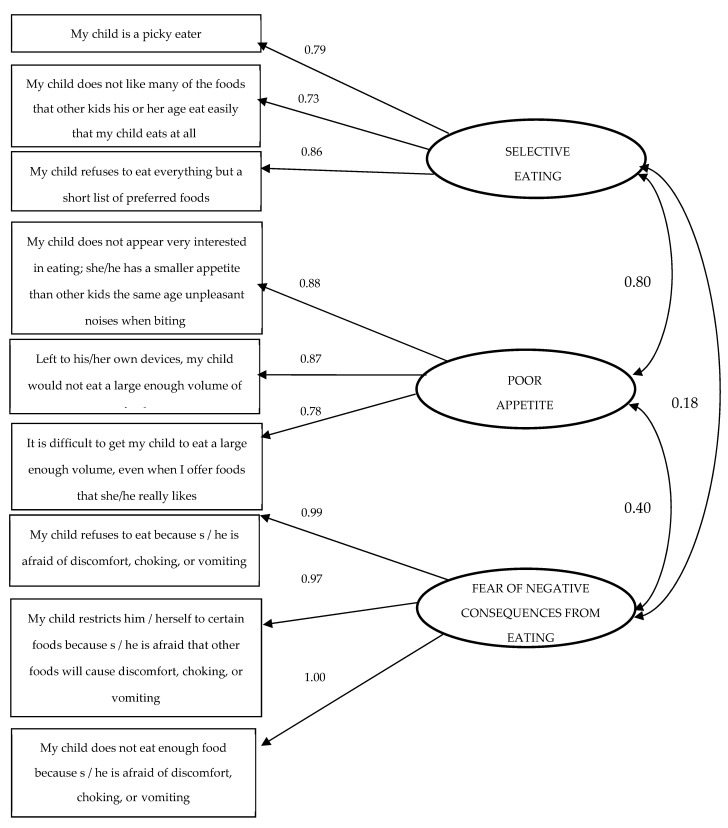
Confirmatory analysis showing the three factors of NIAS-PR.

The analysis of the statistics for the NIAS-PR test showed that the reliability of the entire test reached the Cronbach’s Alpha value = 0.88 and the mean correlation between items 0.52 (from 0.45 to 0.79). The confirmatory analysis of the NIAS-PR test confirmed its univariate structure (Figure 2).

The determination of the result, which is a diagnostic criterion for ARFID, was carried out by means of the analysis of the results of mean values and the correlation of the subscales with the total score of the ARFID-Q-PR. Pearson’s r correlations (Table 3) showed that the ARFID-Q-PR subscales: “attitude to food” (*r =* 0.81) and “justification for restriction” (*r* = 0.77) significantly correlated with the sum of the questionnaire. On the other hand, the “somatic condition” subscale in this group of respondents does not show such a relationship with the sum of the ARFID-Q-PR (*r* = 0.14). Importantly, somatic health problems may or may not be the result of an eating disorder. In order to avoid a situation in which, for example, a somatically ill person would obtain high results on the ARFID-Q-PR scale, which are not a consequence of an eating disorder, two ARFID criteria had to be established: the first condition sine qua non for the sum of the subscales “attitude to food” and “justification for restriction”, and the second—for the grand total of ARFID-Q-PR (sum of three subscales):

(a) criterion for the sum of the subscales “attitude to food” and “justification for restriction”: the mean for the sum of these subscales was M = 18.26 (SD = 6.00). The score indicating symptoms of ARFID was determined by summing up the mean value and 1 SD (25 points). There were 23 people who achieved this result in the sample (13.77%).
(b) criterion for the ARFID-Q-PR overall score (sum of the subscales “attitude to food, justification for restriction” and “somatic condition”): the mean for the overall ARFID-Q-PR score was M = 23.83 (SD = 5.72). The score that indicates symptoms of ARFID was determined by summing up the mean value and 2 SD (35 points). There were 6 (3.59%) people in the sample who achieved this result.

## 4. Discussion

The objective of the presented study was to assess the psychometric properties of the Avoidant/Restrictive Food Intake Disorder Questionnaire—Parents Report (ARFID-Q-PR) that evaluates mothers’ perspectives on their young children with ARFID (aged 2 to 10 years old) and to confirm the three-factor structure of the Polish version of the Nine Items Avoidant/Restrictive Food Intake Disorder Screen—Parents Report (NIAS-PR).

The reliability of the ARFID-Q-PR proved to be satisfactory (Cronbach’s alpha = 0.84). Our findings indicated three factors related to ARFID. The first, “attitude to food” (Cronbach ‘s alpha = 0.76) refers to revealing the tendency to avoid/limit food intake, i.e., eating too small portions/volumes in relation to the body’s needs. It is the basic criterion determining the diagnosis of ARFID. The analysis of the results based on case studies indicates that food restrictions appear as early as early childhood but are often recognized much later [14,42]; food products that are excluded from the menu vary widely (restricting eating bread vs. eating only bread, eating only packaged, highly processed snacks [43]). Food intake seems to have nothing to do with its caloric content, and people with ARFID do not show concern about body weight and appearance [1]. Recent research [33] has demonstrated that 4–7-year-old Japanese children with ARFID displayed more problems associated with restrictive-type eating and nutritional intake. They more often presented selective eating and a decreased appetite. In addition, they ate less due to being sensitive to satiety and to feeling negative emotions. Furthermore, Eddy and Thomas [42] have emphasized the necessity for assessment of specific psychopathology related to ARFID: food avoidance and restriction (across the five basic categories of fruits, vegetables, protein, dairy, and grains), sensory sensitivity (e.g., avoiding fruits and vegetables), fear of aversive consequences (e.g., choking, vomiting), and lack of interest in eating and food (e.g., eating small portions at most meals, skipping some meals).

The second factor, “justification for restriction” (Cronbach’s alpha = 0.73) is related to establishing reasons for avoiding food, i.e., the primary ARFID subtypes (1. sensitivity to food sensory properties, 2. no interest in food, and 3. fear of the consequences of food intake). The existence of ARFID subtypes is confirmed by many researchers. In retrospective studies on 83 children aged 8–17 [16], four subgroups were distinguished that avoided eating for various reasons. It was observed that apart from a different clinical picture of ARFID, children from different subgroups also differed in the duration of the disorder, mood, comorbidities, age, gender, and symptoms of psychopathology reported by their parents.

Duncombe Lowe et al. [18], based on a study of a clinical sample of 102 people aged 8 to 18 years, proved that about 19% of the respondents showed a lack of interest in food, 16% showed fear of the consequences of eating, nearly 15%—sensory sensitivity, while all ARFID presentations simultaneously appeared in about 10% of the studied sample. Moreover, it turned out that half of the patients met the criteria for more than one symptom of ARFID, and the clinical picture of the disorder was related to age, body weight, and duration of symptoms.

Based on a systematic review of scientific texts on ARFID, Bourne et al. [44] recognized that it is necessary to analyze the heterogeneous presentation of this disorder to better understand the mechanisms that drive avoiding/restricting food, which will contribute to increasing the effectiveness of therapeutic interventions. Additionally Strand et al. [45] conducted an analysis of more than twenty empirical texts as well as case studies on ARFID. On this basis, she considers that the current DSM-5 criteria are not helpful in the accurate diagnosis of the disorder and suggests their clarification, i.a., regarding the subtypes of ARFID.

In the current form of the ARFID-Q-PR questionnaire, items related to sensory sensitivity are dominant; therefore, the tool requires further work in order to identify the possible full range of symptoms of all ARFID subtypes.

The third factor of ARFID-Q-PR, “somatic condition” (Cronbach’s alpha = 0.67) refers to the medical consequences of malnutrition, which are just beginning to be outlined in the literature because only recently, research on complications of ARFID has been conducted [43]. According to Nitsch et al. [46], pediatric patients with ARFID may have countless physical ailments, e.g., from the digestive system, electrolyte disturbances, less often bradycardia, low bone density. Yule et al. [47] retrospectively analyzed 76 cases of patients from 2.5 to 17 years old. They proved that the result of malnutrition resulting from avoiding food intake is a deficiency of vitamin C, A, thiamine, vitamin B-12, and D.

In the ARFID-Q-PR questionnaire, items of the third subscale refer to such signs of deterioration of the child’s somatic health (e.g., syncope, somnolence) that are available for observation by caregivers.

There are several limitations to the presented study. First, due to the cross-sectional design, we are unable to establish the test–retest reliability of the ARFID-Q-PR. Second, the sample from the female population (our study relied on maternal report) limits generalizability. Future studies should investigate the psychometric priorities of the ARFID-Q-PR among fathers of children with ARFID and rely upon empirically validated measures. Examination of clinical features of young children diagnosed with ARFID would be needed. Third, the nutrient and caloric intake of young children was not assessed; thus, we cannot evaluate nutritional profile and food variety in this group. In the future study, the food-based assessment methods of dietary intake among young children with ARFID should be taken into consideration.

## 5. Conclusions

Our results showed that, from the mother’s perspective, children in the general population scored lower on ARFID symptoms than children with developmental and mental disorders. This suggests that ARFID-Q-PR completed by mothers has the potential to differentiate children with ARFID associated with a co-occurring mental disorder from typically developing children. Thus, it may serve as a screening tool used by mothers in the identification of ARFID among young children.

In subsequent studies, the interview should include questions about the co-existing disorders in the child and its primary feeder and about eating problem among parents. To the best of our knowledge, no study has been conducted to investigate mental disorders among parents of children with ARFID, specifically considering the subtypes of ARFID. However, a recent study [48] has found that mothers of children with ARFID reported higher levels of health-related quality of life regarding pain and higher levels of distress regarding cognitive problems compared to those of healthy children.

ARFID has been found to be presented as a parent-related feeding disorder or as a child-related eating disorder [19]. A recent study [19] has demonstrated that both children and their parents exhibited the higher rates of eating problems and reported enacting more maladaptive feeding patterns compared to children and parents in the control group. It has been suggested [49] that ARFID is characterized by maladaptive parental feeding patterns. In the future study, we are planning further work on the tool to include items indicating all ARFID subtypes, taking into account the nutritional behavior of both children and their parents.

## Figures and Tables

**Table 1:** Selected tools for the assessment of symptoms of Avoidant/Restrictive Food Intake Disorder (ARFID) and related disorders.

Assessment Tool	Authors	Sample	Symptoms	Reliability
Eating Disorder Assessment for *DSM-5* (EDA-5)	Sysko et al., 2015 [10]	Adults	Pica, RD ARFID (all presentations)	κ = 0.87
Pica, ARFID, and Rumination Disorder Interview (PARDI)	Bryant-Waugh et al., 2018, 2019	2 years—adult	Pica, RD ARFID (all presentations)	α (for subscales) = 0.77 to 0.89
Eating Disturbances in Youth-Questionnaire (EDY-Q)	Hilbert & van Dyck, 2016	8–13 years	Pica, RD ARFID (all presentations)	α = 0.62
Nine-item ARFID Screen (NIAS)	Zickgraf & Ellis, 2018	Adults	ARFID (all presentations)	α = 0.79

Source: based on Sysko et al. [10].

**Table 2:** NIAS-PR: English and Polish version.

	English Version of the NIAS-PR	Polish Version of the NIA-PR
1	My child is a picky eater	Moje dziecko jest wybredne
2	My child doesn’t like many of the foods that other kids his or her age eat easily	Moje dziecko nie lubi większości potraw, które jedzą jego rówieśnicy
3	My child refuses to eat everything but a short list of preferred foods	Lista produktów, które lubi i zjada moje dziecko jest krótsza, niż lista produktów, których nie zjada
4	My child does not appear very interested in eating; s/he has a smaller appetite than other kids the same age	Moje dziecko nie jest bardzo zainteresowane jedzeniem; Wydaje mi się, że ma mniejszy apetyt niż jego rówieśnicy
5	Left to his/her own devices, my child would not eat a large enough volume of food	Pozostawione samemu sobie, moje dziecko nie zjadłoby wystarczająco dużej ilości jedzenia
6	It is difficult to get my child to eat a large enough volume, even when I offer foods that s/he really likes	Pozostawione samemu sobie, moje dziecko nie zjadłoby wystarczająco dużej ilości jedzenia
7	My child refuses to eat because s/he is afraid of discomfort, choking, or vomiting	Moje dziecko unika jedzenia, ponieważ boi się dyskomfortu w żołądku, duszenia się lub wymiotów
8	My child restricts him/herself to certain foods because s/he is afraid that other foods will cause discomfort, choking, or vomiting	Mojemu dziecku trudno jest jeść wystarczająco dużą objętość pokarmu, nawet gdy oferuję mu produkty, które naprawdę lubi
9	My child does not eat enough food because s/he is afraid of discomfort, choking, or vomiting	Moje dziecko nie je wystarczającej ilości jedzenia, ponieważ boi się dyskomfortu, zadławienia lub wymiotów

Note: NIAS-PR: Nine Items Avoidant/Restrictive Food Intake Disorder Screen—Parent Report.

**Table 3:** The ARFID Questionnaire—Parent Report (ARFID-Q-PR): Polish and English version.

	Polish Version of the ARFID-Q-PR	English Version of the ARFID-Q-PR
1	Moje dziecko nie jest zainteresowane jedzeniem	My child is not interested in eating
2	Moje dziecko nie je niektórych potraw, bo jego zdaniem wydają nieprzyjemne dźwięki podczas gryzienia	My child does not eat certain foods because he thinks they make unpleasant noises when biting
3	Moje dziecko ma problemy jelitowe (np. zaparcia, biegunki, itp.)	My child has intestinal problems (e.g., constipation, diarrhea, etc.)
4	Inne osoby (np. rodzina, wychowawca w przedszkolu) pilnują, aby moje dziecko w ogóle jadło	Other people (e.g., family, kindergarten teacher) make sure that my child eats at all
5	Moje dziecko nie je niektórych potraw, bo jego zdaniem brzydko pachną	My child does not eat some foods because he thinks they smell bad
6	Moje dziecko ma problemy gastryczne (np. bóle żołądka, kolki, wzdęcia)	My child has gastric problems (e.g., stomach pain, colic, flatulence)
7	Moje dziecko je, o ile porcje są małe	My child eats if the portions are small
8	Moje dziecko nie je niektórych potraw, bo jego zdaniem mają nieprzyjemną konsystencję/fakturę	My child does not eat some dishes because he thinks they have an unpleasant consistency/texture
9	Moje dziecko szybko się męczy	My child gets tired quickly
10	Moje dziecko twierdzi, że nie czuje głodu/nie jest głodne	My child says he is not hungry
11	Moje dziecko zapada na infekcje (przeziębienie, katar itp.)	My child has infections (colds, runny nose, etc.)
12	Moje dziecko wypluwa jedzenie	My child spits out food
13	Moje dziecko unika sytuacji związanych z jedzeniem	My child avoids eating situations
14	Moje dziecko jest senne/słabe/bez energii	My child is sleepy/weak/without energy

**Table 4:** Pearson’s *r* correlations subscales for ARFID-Q-PR with NIAS-PR and age, gender, chronic diseases, BMI, means, and standard deviations of individual variables.

	1	2	3	4	5	6	7	8	9	10	11	12
1. ARFID-Q-PR Attitude to food	-											
2. ARFID-Q-PR Justification for restriction	0.58 ***	-										
3. ARFID-Q-PR Somatic condition	−0.26 **	−0.28 ***	-									
4. ARFID-Q-PR Total	0.81 ***	0.77 ***	0.17 *	-								
5. NIAS-PR Selective eating	0.61 ***	0.58 ***	−0.18 *	0.61 ***	-							
6. NIAS-PR Poor appetite	0.64 ***	0.56 ***	−0.20 *	0.60 ***	0.67 ***	-						
7. NIAS-PR Fear of negative consequences from eating	0.28 ***	0.25 **	−0.37 ***	0.13	0.13	0.37 ***	-					
8. NIAS-PR Total	0.68 ***	0.62 ***	−0.30 ***	0.61 ***	0.84 ***	0.90 ***	0.55 ***	-				
9. Age	−0.12	0.15	−0.13	−0.05	<0.01	0.10	0.13	0.09	-			
10. Gender	−0.17 *	−0.04	0.16 *	−0.05	−0.09	−0.04	−0.03	−0.07	0.19 *	-		
11. Chronic diseases	−0.01	0.05	−0.11	−0.03	0.14	−0.03	0.19 *	0.12	0.17 *	−0.06	-	
12. BMI	−0.10	−0.08	−0.07	−0.14	−0.07	<0.01	0.07	−0.01	0.15	0.08	0.09	-
M.	9.75	8.51	5.56	23.83	6.01	4.17	1.20	11.38	5.77	-	-	15.83
SD	3.63	3.11	2.81	5.72	4.19	3.73	2.70	8.40	2.47	-	-	2.26

Note: *** *p* < 0.05; ** *p* < 0.01; * *p* < 0.001.

**Table 5:** EFA ARFID-Q-PR factor analysis (N = 167).

Item	Factor 1: Attitude to Food	Factor 2: Justification Restrictions	Factor 3: Fitness Somatic
1. My child is not interested in eating	0.65	−0.07	0.19
2. My child does not eat certain foods because he/she thinks they make unpleasant noises when biting	0.00	0.32	0.52
3. My child has intestinal problems (e.g., constipation, diarrhea, etc.)	0.24	0.64	0.00
4. Other people (e.g., family, kindergarten teacher) make sure that my child eats at all)	0.70	0.16	0.26
5. My child does not eat some dishes because he/she thinks they smell bad	0.27	0.22	0.72
8. My child does not eat some dishes because he/she thinks they have an unpleasant consistency/texture	0.24	0.16	0.80
9. My child gets tired quickly	0.00	0.57	0.27
10. My child says he or she is not hungry/hungry	0.52	0.05	0.45
11. My child has infections (cold, runny nose, etc.)	0.37	0.44	−0.09
12. My child spits out food	0.60	0.40	−0.03
13. My child avoids eating situations	0.76	0.23	0.19
14. My child is sleepy/weak/without energy	0.14	0.66	0.27
6. My child has gastric problems (e.g., stomach pain, colic, flatulence)	−0.03	0.71	0.25
7. My child eats as long as the portions are small	0.51	−0.01	0.54
Out condition	20.72	20.30	20.28
Participation	0.19	0.16	0.16

Note: EFA—exploratory factor analysis; numbers represent raw factor loadings; N—the size of sample.

**Table 6:** EFA NIAS-PR factor analysis (N = 167).

Item	Factor 1 Selective Eating	Factor 2 Poor Appetite	Factor 3 Fear of Negative Consequences from Eating
My child is a picky eater	0.83	−0.04	0.28
2. My child doesn’t like many of the foods that other kids his or her age eat easily	0.84	0.04	0.18
3. My child refuses to eat everything but a short list of preferred foods	0.77	0.14	0.39
4. My child does not appear very interested in eating; s/he has a smaller appetite than other kids the same age	0.48	0.22	0.73
5. Left to his/her own devices, my child would not eat a large enough volume of food	0.41	0.24	0.76
6. It is difficult to get my child to eat a large enough volume, even when I offer foods that s/he really likes	0.16	0.18	0.90
7. My child refuses to eat because s/he is afraid of discomfort, choking, or vomiting	0.05	0.98	0.14
8. My child restricts him/herself to certain foods because s/he is afraid that other foods will cause discomfort, choking, or vomiting	0.04	0.99	0.14
9. My child does not eat enough food because s/he is afraid of discomfort, choking, or vomiting	0.04	0.99	0.14
Out condition	2.44	3.07	2.25
Participation	0.27	0.34	0.25

**Table 7:** Analysis of the reliability of the ARFID-Q-PR (N = 167).

Factor	Item	Correlation	Alpha When Removed
Attitude to food Cronbach’s alpha = 0.76	1. My child is not interested in eating	0.46	0.74
	4. Other people (e.g., family, kindergarten teacher) make sure that my child eats at all	0.58	0.69
	10. My child says he or she is not hungry/hungry	0.48	0.73
	12. My child spits out food	0.45	0.74
	13. My child avoids eating situations	0.71	0.66
Justification for the restriction Cronbach’s alpha = 0.73	2. My child does not eat certain foods because he/she thinks they make unpleasant noises when biting	0.34	0.77
	5. My child does not eat some dishes because he/she thinks they smell bad	0.64	0.61
	8. My child does not eat some dishes because he/she thinks they have an unpleasant consistency/texture	0.70	0.57
	7. My child eats as long as the portions are small	0.46	0.72
Somatic condition Cronbach’s alpha = 0.67	3. My child has bowel problems (e.g., constipation, diarrhea, etc.)	0.42	0.60
	6. My child has gastric problems (e.g., stomach pain, colic, flatulence)	0.48	0.57
	9. My child gets tired quickly	0.39	0.62
	11. My child has infections (cold, runny nose, etc.)	0.30	0.66
	14. My child is sleepy/weak/without energy	0.52	0.57

Note: N—the size of sample.

**Table 8:** Analysis of the reliability of the NIAS-PR (N = 167).

Factor	Item	Correlation
Selective eating Cronbach’s alpha = 0.84	1. My child is a picky eater	0.84
	2. My child doesn’t like many of the foods that other kids his or her age eat easily	0.85
	3. My child refuses to eat everything but a short list of preferred foods	0.78
Poor appetite Cronbach’s alpha = 0.88…	4. My child does not appear very interested in eating; s/he has a smaller appetite than other kids the same age	0.72
	5. Left to his/her own devices, my child would not eat a large enough volume of food	0.76
	6. It is difficult to get my child to eat a large enough volume, even when I offer foods that’s/he really likes	0.90
Fear of negative consequences from eating Cronbach’s alpha = 0.99…	7. My child refuses to eat because s/he is afraid of discomfort, choking, or vomiting	0.98
	8. My child restricts him/herself to certain foods because s/he is afraid that other foods will cause discomfort, choking, or vomiting	0.97
	9. My child does not eat enough food because s/he is afraid of discomfort, choking, or vomiting	0.98

**Table 9:** Confirmatory analysis of CFA ARFID-Q-PR and NIAS-PR (N = 167).

	CMIN	*p*	CMIN/df	RMSEA	AGFI	TLI	CFI
ARFID-Q-PR (three factor model)	81.22	0.114	1.21	0.036	0.902	0.971	0.978
NIAS-PR (three factor model)	29.63	0.197	1.24	0.038	0.930	0.995	0.997

Note: CFA—Confirmatory Factor Analysis; N—the size of sample; SEM—Structural Equation Modeling; CMIN—the value of chi square statistic; *p*—statistical significance; CMIN/df—Chi square statistic and df are the degrees of freedom; RMSEA—Root Mean Square Error of Approximation; AGFI—χ2 Adjustment Goodness of Fit Index; TLI—Tucker—Lewis Index CFI, Comparative Fit Index.

## Data Availability

The dataset used during the current study are available from the corresponding author on reasonable request.
